# COVID-19 and Adapting to the New Normal: Lessons Learned for Peacebuilding

**DOI:** 10.1177/15423166211052832

**Published:** 2021-11-22

**Authors:** Serena Clark, Claudio Alberti

**Affiliations:** 8798 Maynooth University, Maynooth, Ireland; 8809 Trinity College Dublin, Dublin, Ireland

**Keywords:** localization, local turn, adaptive peacebuilding, COVID-19, digital peacebuilding

## Abstract

The World Health Organization (WHO) declared the novel Coronavirus (SARS-CoV-2; Covid-19) a pandemic on 11 March 2020. Unlike preceding highly contagious diseases that brought the threat of global instability this century, such as SARS-CoV, Zika virus (ZIKV), Swine flu (H1N1), and Avian flu (H5N1), Covid-19, governments across the world introduced strict measures and interruptions to daily life incomparable in living memory. Overnight, countries closed schools, higher education institutions, workplaces and shut down borders – this left people scrambling to adapt, including those implementing peacebuilding interventions. In this unprecedented situation, peacebuilding organisations have worked, responded, and adapted to the new normal. These new dynamics have created both challenges and opportunities for peacebuilding. This article documents the experiences of peacebuilders during the pandemic, making sense of changing conditions, challenges and opportunities they faced. It explores two key questions. How have peacebuilding organisations adapted during COVID-19? Has COVID-19 contributed to the move to local ownership of peacebuilding or localisation? It addresses these questions by engaging with peacebuilding organisations across different geographical regions through an online survey and key informant interviews. The main results focus on localisation, digital adaptation and funding strategy and administration challenges.

## Introduction

The World Health Organization (WHO) declared the novel coronavirus (COVID-19) a pandemic on 11 March 2020. Unlike other highly contagious diseases that threatened to undermine global stability this century, such as SARS-CoV, Zika virus (ZIKV), Swine flu (H1N1), and the Avian flu (H5N1), COVID-19 could not be controlled. Overnight, countries closed their borders, announced national lockdowns and shelter-in-place orders and closed schools, higher education institutions and workplaces. The strict lockdown measures introduced by governments worldwide interrupted daily life in a way incomparable to anything in living memory. People were left scrambling to adapt, including those implementing peacebuilding interventions. It is this phenomenon of how peacebuilders responded and adapted during COVID-19 that this article explores.

### The implication of COVID-19 in fragile and conflict-affected countries

COVID-19 and the global response have had severe implications for conflict-affected countries, in many cases exacerbating existing inequalities, food insecurity, gender-based violence, community violence, stigmatisation, unemployment and human rights violations (Conducive Space for Peace, 2020, p. 2; Peace Direct, 2020, p. 3; United Nations World Food Programme, 2020a, p. 8). As of June 2021, the pandemic has killed over four million people (John Hopkins University, 2021) and continues to impact healthcare systems, the economy and governance. Though the pandemic has affected all world regions, it has significantly impacted the most impoverished and vulnerable. With the pandemic interrupting peacebuilding processes and at times reigniting or fuelling conflict, the United Nations (UN) Secretary-General called for a global ceasefire. Of the 43 countries recording significant levels of organised violence, only ten saw actors that ‘welcomed’ the ceasefire call, 21 ignored it altogether, and many quickly dissolved in places like Yemen, Columbia and the Philippines. The UN World Food Programme chief said 2021 would be ‘the worst humanitarian crisis year since the beginning of the United Nations’ (United Nations World Food Programme, 2020b).

Physical distancing and blaming ‘the other’ for the spread of COVID-19 has also exacerbated existing hostilities between conflicting groups (Peace Direct, 2020, p. 3). Peacebuilding programs and dialogues were stopped in countries such as South Sudan, Somalia, Kenya, Cameroon and the Democratic Republic of the Congo, and in Columbia, the peace process stagnated (Peace Direct, 2020, p. 3). In Yemen, the pandemic continues to worsen one of the world's worst humanitarian crises. In Lebanon, a country already plagued by economic crises, the pandemic has become a ‘crisis within a crisis’ (Abou-Zahr, 2020), with half the population living in poverty. In other countries, such as Tanzania, the government continues to deny the existence of COVID-19 altogether (Oduor, 2021).

### Adapting to a new normal

Peacebuilding organisations have been working, responding, and adapting to the new normal within this unprecedented situation. The long-term effects of the pandemic on existing armed conflict and the risks of intensified levels of violence are not yet known. However, the short- and medium-term consequences for local and international peacebuilders is clear (Conducive Space for Peace, 2020, p. 2; Desmidt & Neat, 2020, pp. 1–5). The implications of this crisis will keep evolving, creating varied and interconnected challenges and opportunities for all stakeholders in the peacebuilding field. As a result, it is essential to document and understand these changes to address present and long-term implications for peacebuilding operations (Conducive Space for Peace, 2020, p. 2).

### The study

This article documents the experiences of peacebuilders during the pandemic, making sense of changing conditions, challenges and opportunities they faced. It explores two key questions. How have peacebuilding organisations adapted during COVID-19? Has COVID-19 contributed to the move to local ownership of peacebuilding or localisation? This article addresses these questions, presenting research findings from a study engaging 153 peacebuilding organisations across 41 geographical areas in an online survey and 27 peacebuilders from 15 countries in semi-structured interviews. A deeper understanding of the pandemic's impact on peacebuilding operations is essential for considering best practices for conceptualising and implementing interventions during crises in the future and post-pandemic.

## COVID-19 pandemic: localising, digitalising and reshaping funding mechanisms

The COVID-19 outbreak forced peacebuilding and development actors to quickly adapt to a ‘new normal’ and reorganise their work to continue operating in this new context. Some of the sudden adopted changes initially meant to be temporary seem to be destined to remain in place and have the potential to reshape the sector in the medium to long term. This study found three main areas that are and will be affected by these changes: localisation, the use of digital solutions, and funding strategy and administration. The three areas mutually influence each other, as the need to implement locally led and digital solutions requires rethinking how the funding system for the sector currently works.

### COVID-19: An opportunity to rethink peacebuilding

The COVID-19 pandemic offers an opportunity to rethink the conceptualisation and implementation of peacebuilding. There is consensus around the complexity and uncertain nature of peacebuilding operations alongside the need to be locally driven (de Coning, 2018, p. 309; de Coning, 2020, p. 851; Paffenholz, 2009, p. 868). Despite this consensus, these operations remain highly technical and implemented using specific parameters and pre-established tools across different contexts. In December 2013, the events that led to violent conflict in South Sudan and the destabilisation and collapse of society in Syria and Yemen showed how unpredictable the non-linear and emergent behaviours of complex social systems are, especially during times of conflict. These examples show the need for tools, such as conflict analysis and needs assessments, to be ongoing iterative processes rather than predefined steps in a determined-design program cycle (de Coning, 2018, p. 309). Against the backdrop of the pandemic, peacebuilding has become even more complex and brings to the forefront the debate around models of localisation and adaptation. Various studies highlight how COVID-19 impacted the health sector, contributed to exacerbating conflict dynamics, and increased socioeconomic divides across the globe, making it a potential threat to peace and security (Conducive Space for Peace, 2020, p. 2; Desmidt & Neat, 2020; Interpeace, 2020; International Alert, 2020).

Against the backdrop of the pandemic, peacebuilding has become even more complex and brings to the forefront the debate around models of localisation and adaptation.

Additionally, many local peacebuilding organisations face challenges implementing projects during the pandemic because of a shift in allocating funds from implementing peace interventions to COVID-19 emergency response (Peace Direct, 2020, p. 6) or reducing funds themselves (Conducive Space for Peace, 2020, p. 2). The Gross National Income figures of donor countries have been shrinking with the pandemic, and as a result, Official Development Assistance (ODA) is likely to decrease. Development Initiative presents a scenario where there could be a reduction in ODA of $25 billion by 2021 (Conducive Space for Peace, 2020, p. 8; Development Initiatives, 2020, p. 3). Peacebuilding can no longer be understood as a standalone process. These recent challenges have demonstrated the necessity to incorporate peacebuilding into established development and humanitarian interventions (Interpeace, 2020, pp. 5–6).

### The challenges of peacebuilding in the contemporary era

The global peacebuilding system incorporates many inter-connected organisations, and development and humanitarian interventions are composed of many different actors. Each actor has experienced various stresses related to COVID-19 that impact the others. Bilateral donors have money and power. However, they have capacity constraints for managing smaller grants and are under pressure from donor constituencies to demonstrate the impact of development assistance and justify the significance for donor countries. Though encouraged to free resources for the pandemic response, these donors often have a limited understanding of the situation on the ground and the needs of local peacebuilders, making it challenging to reprioritise funds (Conducive Space for Peace, 2020, p. 6).

There are also intermediaries, such as international non-governmental organisations (INGOs) and the UN. INGOs usually have long-standing relationships with local peacebuilders and donors, therefore serving an essential intermediary role. They can support local organisations in many ways, and because they often have access to unrestricted funds from private donors, they can support risk-prone and innovative engagement. Though INGOs can understand needs and foster solutions, they depend on donors that may be inflexible to evolving situations. Moreover, the organisational structures of larger INGOs are not easily transformed to meet the demands of changing conditions. During COVID-19, these organisations have experienced a deficit in funding and access to conflict-affected countries (Conducive Space for Peace, 2020, p. 6). The UN has access to information on peacebuilding needs across all levels of conflict and communicates regularly with bilateral donors and INGOs. However, the UN has limited authority over bilateral donors and, when trying to enhance civil society support, they may face resistance by state actors. The lack of access in conflict-affected contexts, the limited interest of governments in peacebuilding processes and the absence of structures to support local peacebuilders may restrict the UN's abilities further (Conducive Space for Peace, 2020, p. 6).

Though INGOs can understand needs and foster solutions, they depend on donors that may be inflexible to evolving situations.

### A new push for localisation

Larger national non-governmental organisations (NGOs) can support local organisations and actors but obtaining funding is increasingly challenging, creating competition and decreasing effective collaboration in shrinking civic spaces. More prominent national NGOs tend to know how to work with local peacebuilders and within the donor space. However, they face reduced access to local contexts, a deficit in funds, interruptions in activities, and fewer opportunities to apply for grants. Local NGOs often have established relationships with local communities and can address peacebuilding needs holistically. However, accessing funds and meeting the requirements of donors can be a problem. These organisations face needs to adapt interventions, insufficient digital capacities, and uncertainty in their future, all compounded by financial difficulties (Conducive Space for Peace, 2020, p. 6). With international organisations unable to be on the ‘frontlines’ due to the pandemic, local organisations have become the driving force behind peacebuilding initiatives on the ground (Conducive Space for Peace, 2020, p. 2). The pandemic has created an environment where local organisations play a leading role in peacebuilding operations (Conducive Space for Peace, 2020, p. 2; Peace Direct, 2020, pp. 2–6). This arrangement shows what localisation looks like in practice (de Coning, 2020; Peace Direct, 2020). Over the past two decades, ‘local turn’ and ‘local ownership’ have been at the forefront of academic debate. Many scholars advocate engaging and empowering conflict-affected communities in peacebuilding processes (Funk, 2012, p. 396; Paffenholz, 2009, p. 859). However, the realisation of including local communities in peacebuilding processes has rarely occurred. Instead, local ownership remains vaguely defined and is often only used as a ‘buzzword’ (Richmond, 2012, p. 354). Meanwhile, the local turn is regularly used to legitimise and implement an externally conceived liberal agenda (Vogel, 2016, p. 14). As travel restrictions prevent international actors from accessing the countries where they operate, they have turned to local organisations to take greater ownership over peacebuilding interventions. With this shift, local actors are no longer simply implementation partners but taking on the role of their external peacebuilding counterparts. For this change in ownership to continue in the medium to long term, the existing dynamics between donors, international organisations, and Civil Society Organizations (CSOs) requires reconsideration. Reshifting these relationships will move the role of local actors from solely implementers to collaborators in designing interventions and providing support to domestic political processes (de Coning, 2020, p. 851). With this newfound ownership, local peacebuilding organisations are learning and adjusting to the new situation to address emerging needs (Peace Direct, 2020, p. 4), beginning the process of ‘passive adaptation’ (Williams, 2011, p. 1373). Though this was the natural step, it highlighted the limits of the organisational capacity of peacebuilding actors to respond in a fast-changing environment (Peace Direct, 2020, p. 5). This experience is an opportunity to advocate for ‘active adaption,’ implying a move beyond modifying interventions to address the new normal but conceiving and developing interventions based on the principle of experimentation and selection as envisioned by the Adaptive Peacebuilding model (de Coning, 2020, p. 851).

As travel restrictions prevent international actors from accessing the countries where they operate, they have turned to local organisations to take greater ownership over peacebuilding interventions.

### Peacebuilding in the digital space

The possibility for local actors to experiment with new approaches is moving peacebuilding from the ‘real world’ to the digital space (International Alert, 2020, p. 5). Peacebuilding operations are traditionally highly dependent on face to face interactions. In-person activities are integral to the trust-building process between communities, implementers, and different groups (Alberti & Clark, 2020; International Alert, 2020, pp. 5–7). On the one hand, digital solutions such as social media can increase the engagement of young people in peacebuilding related work. On the other hand, digital space can reduce the opportunities for intergroup exchanges that promote dialogue and peaceful coexistence (International Alert, 2020, p. 8). It can further marginalise conflict-affected communities in rural areas, favouring elite capture (International Alert, 2020, p. 7; Peace Direct, 2020, p. 3), meaning more engagement with people living in urban areas. These areas often have better digital infrastructure and fewer economic barriers to accessing broadband and phones (Alberti & Clark, 2020). Another critical aspect to consider with using digital solutions in peacebuilding interventions is protection issues for participants. Dialogue sessions often touch upon sensitive and controversial issues. If confidentiality is not ensured, participants may be exposed to threats and retaliation (International Alert, 2020, p. 6) from conflict actors, spoilers and national authorities.

Though there are already a few studies on the effects of COVID-19 on peacekeeping(Carnegie & Carson, 2021; de Coning, 2020) and international development (Brown, 2021; Gavas & Pleeck, 2021), there is limited knowledge of how the pandemic affects the peacebuilding sector. Some think tanks and NGOs have reported initial data and findings (Conducive Space for Peace, 2020; de Coning, 2020; Interpeace, 2020; Peace Direct, 2020) in the first phase of the pandemic in the first half of 2020. This research expands on these reports, contributing to the discussion. It offers valuable insights into how the pandemic is changing the peacebuilding, humanitarian and development sectors in the present and future.

## Methodology

### Design

This study uses a mixed-method approach inclusive of an online survey, semi-structured interviews, and deductive thematic analysis. This approach allows for the generation of rich information about participants’ experiences and the quantification of key data. The authors developed the survey and semi-structured interviews based on their academic and professional experience in the United Nations. The survey and semi-structured interview schedule are in Appendix 1. The survey gathered both quantitative and qualitative data. It was distributed in English, French and Spanish, and the interviews were conducted in these three languages. These languages were selected as they allowed access to different regions and are the languages the authors are fluent in and could use for conducting semi-structured interviews.

Due to COVID-19, researchers used online platforms for the interviews. This approach was used to prevent person-to-person contact and because of travel restrictions and lockdowns. Discussions took place over Zoom, WhatsApp, Skype and via email depending on access to technologies by each interviewee. Before interviews, participants were informed of the information in the consent forms, such as anonymity and the ability to leave the study at any time up until publication. Once they agreed to participate, an interview was carried out. If the participant was comfortable, the interview was recorded and transcribed. For interviews where participants were not comfortable with recording, the researchers took notes during the interview. To ensure the anonymity of respondents, the quotes included in this article are not attributed to the interviewees.

This study received ethical approval from the Social Research Ethics Subcommittee, Maynooth University (2428355).

### Participants and recruitment

Survey respondents were identified using Peace Direct's peace organisations lists and the researchers’ professional networks. Additionally, internet searches were conducted to identify peace organisations when countries were not listed on Peace Direct, such as the United States and the United Kingdom. A total of 153 organisations from 41 geographical areas responded to the survey. The term geographical area is used instead of countries because some are disputed territories (See Table 1). Though the researchers sought a diverse sample set, participation depended on the willingness of people to participate in the study. As a result, most participants came from secular local peacebuilding organisations.

**Table 1.** Geographical areas included in the study

Afghanistan	Algeria	Bangladesh	Rwanda	France
Chad	Chile	China	Cote d’Lvoire	Iraq
Georgia	Germany	India	Srinagar	Central African Republic
Malawi	Ireland	Jerusalem	Lebanon	Democratic Republic of the Congo
Kazakhstan	Nigeria	Mexico	Nepal	District of Jammu & Kashmir State
Niger	Pakistan	Ukraine	Senegal	Mozambique
Sri Lanka	Uganda	United Kingdom	Australia	Bosnia & Herzegovina
Yemen	Zimbabwe	Sierra Leone	Burundi	United States of America
Argentina				

Additionally, the study aimed to represent each respondent in the findings equally; however, some interviewees were more prolific than others. The researchers also acknowledge that various regions were impacted by the pandemic to different degrees when the survey and interviews were carried out, potentially influencing the sample set and responses. For example, when the survey was conducted, the infection rate was significantly higher in Latin America than in Africa. Despite the lower infection rates, African countries were still affected by the pandemic. The closure of borders greatly impacted their already fragile economies, exacerbating some of the existing determinants of fragility. Additionally, misinformation campaigns around the COVID-19 outbreak fueled tensions at the community level, especially in hard-to-reach communities, creating challenges for external actors to work on development and peacebuilding interventions.

Forty-seven per cent of respondents were from national non-governmental organisations (NGOs), 2 per cent from international NGOs, 31 per cent from CSOs, 9 per cent were activists, 2 per cent from academia, 1 per cent from Multilateral Organisations, and 8 per cent were from other entities. Organisations were working on projects in community dialogue, human rights, education, child protection, social protection, livelihoods, the rule of law, mediation, disarmament, demobilisation and reintegration (DDR), employment, health, infrastructure, and security sector reform.

In the survey, participants were asked if they would be willing to be contacted to participate further in the research and asked to provide an email address. Following the survey, the researchers reached out to the respondents who provided an email address. Of those, 27 agreed to be interviewed. The researchers conducted 27 qualitative semi-structured interviews with willing respondents to the survey from 15 different countries. These countries include France, Uganda, Northern Ireland, the United States, Sri Lanka, Australia, Chile, Argentina, Nigeria, the Democratic Republic of the Congo, Chad, Burundi, Senegal, Lebanon and Rwanda. Interviews were conducted in English and French.

### Data Analysis

The quantitative data collected in the survey was analysed and quantified using data analysis in Microsoft Excel. The interview data and qualitative data collected in the study was subjected to deductive thematic analysis. This analysis was guided by the six steps proposed by Braun and Clarke (2008):

1. Familiarisation with the data: Researchers become familiar with the transcripts of the interviews and immerse themselves in the data
2. Generation of initial codes: Researchers start to identify initial codes in the data
3. Identifying themes: Researchers look for common patterns and identify themes
4. Reviewing themes: Researchers review themes to ensure accurate representation of the data
5. Defining themes: Researchers formulate what each theme means in understanding the data
6. Producing the report: Researchers select compelling extract examples, review for final analysis, relate back to the literature and research question, and produce a scholarly report of the analysis.

After conducting and transcribing the interviews, both researchers read through the transcripts several times, individually identifying patterns across the dataset. Following this, the researchers came together to discuss the preliminary codes and form initial potential themes. They then re-analysed the transcripts separately and came together a second time to solidify and define the themes, ensure each theme accurately represented the data, and write up the findings.

Researchers practised reflexivity throughout the research process. This process helped the researchers confront personal positions and biases that may influence the generation of knowledge, the relationship between the researcher, material, and participants and the social implications of the research.

For example, a primary reflexive practice undertaken by the researchers was reflection and discussions with others. As the authors worked themselves at implementing peacebuilding projects during the pandemic, reflecting and exchanging among themselves and other practitioners was crucial to mitigate the risk of the analysis being influenced by their biases based on their personal experience. Reflexivity was especially crucial throughout the coding and analysis process to ensure the findings authentically represented the participants’ lived experiences.

## The Impact of COVID-19 on the Peacebuilding sector

Though many countries are beginning to suppress COVID-19, the pandemic is still growing in some of the most vulnerable countries. Peace organisations and peacebuilders are key players at the forefront of the pandemic, and they are working to help communities facing many challenges. This study found that 78 per cent of participants reported their projects were interrupted due to COVID-19 and public health restrictions. Fifty-three per cent of participants strongly agreed that COVID-19 impacted their projects, and only six per cent said they encountered no interruptions. The main findings focus on localisation, digital adaptation, and funding challenges.

### COVID-19 and the shift to the localisation agenda?

Though there has been an emphasis on encouraging local ownership over the years, this has hardly materialised in practice. Instead of having local peacebuilding organisations endogenously developing and implementing their own solutions, local ownership so far seems to be something built from international actors that empowers to a different extent local organisations. The inability of international actors to reach conflict-affected areas has allowed space to work towards the localisation agenda – supporting locally-led approaches and local agency. As highlighted in an interview with an American organisation working in Ethiopia on mediation and dialogue interventions, their inability to be in the country required local peacebuilders to lead the peacebuilding efforts.

The inability of international actors to reach conflict-affected areas has allowed space to work towards the localisation agenda – supporting locally-led approaches and local agency.

‘*We were working with three cities in the East region bordering the Somali area, and they're mixed ethnically, religiously, and there's lots of conflicts, and our job was to build community relations and trust. In those three cities within each city and between the three cities and we had been there. And we had more scheduled but the pandemic and so what we did instead was we asked them to work only in their own city, ‘cause they weren't travelling either. Send them some ideas and they followed through to greater and lesser extents, and we would meet with them every couple of months to get reports and conversations with them about what they were accomplishing.’* – KI 8, INGO, United States

Donors and INGOs can pause or stop peacebuilding operations, but this is impossible for many local actors. Local actors have continued their activities even when formal projects have been put on hold or discontinued. During COVID-19, peacebuilders are using their increased agency to reconsider priorities and the conceptualisation and implementation of projects, adapting to meet emerging needs. The first organisation below was working on community outreach and dialogue. The second was working with local communities to map memory and creating an online memorial for those that disappeared during a specific conflict. For anonymity, the conflict-setting will not be named.

During COVID–19, peacebuilders are using their increased agency to reconsider priorities and the conceptualisation and implementation of projects, adapting to meet emerging needs.

‘*In two, we were using community dialogues… That one also stopped… I mean the community we are trained on how to form songs, drama, comedy as a way of training people or understanding what's going on…That one also stopped.’* – KI 24, CSO, Uganda

‘*Now the complex realities are affecting the local peacebuilders, and now they're trying their best to adapt to and to face these difficulties.*’ – KI 6, CSO, Lebanon

In having access to local contexts, local peacebuilders were able to do needs assessments to identify emerging demands. On the one hand, local organisations experienced increased pressure to deliver and on the other one, they found themselves fully empowered to develop and implement their own solutions. Some of the organisations changed their operations based on their own needs analysis. Others identified access to basic livelihoods as a significant issue that could exacerbate existing divides and organised basic supplies distributions. Still, other organisations took a different approach. For example, an organisation in Uganda identified an increase in gender-based violence and some minority groups’ exclusion and were pressured to respond to these issues.

‘*Yeah, our operations were constrained. We also saw a surge in so many peacebuilding challenges, gender-based violence being one of them. Gender-based violence, so here is a case where your operations are limited, but then there's a growing need. And also, some of the minority groups in the region experiencing different levels of exclusion. And so there was pressure on us as an organisation*.’ – KI 13, CSO, Uganda

The shift to the local negatively impacted some organisations because of a lack of trust among growing fear. Some organisations became the target of violence or discrimination. Social mobilisers were targeted and considered spreaders. In some cases, social mobilisers belonged to specific ethnic groups. Consequently, ethnic groups were blamed for being spreaders and, in some instances, were threatened and marginalised in their community. Interventions became politicised, which caused problems for programming. In some regions, especially in mining areas, COVID-19 misinformation was used as a tool to limit access of interveners and domestic regulatory authorities so that local elites and non-state actors could benefit from the lack of control and use this opportunity to increase their businesses.

‘*Community members had limited or wrong information about COVID-19. Part of the misinformation was spread by local elites to mobilise the population. Communitymobilisers and their families/group conducting face to face activities despite using all COVID-19 preventive measures became subject of threats and violence causing disruption of activities*’ – KI 20, Burundi

‘*In some areas, some conflict actors benefited from the travel restrictions as they limited opportunities for regulatory authorities and external interveners to access areas in which they were exploiting natural resources. To extend the time they could benefit from this limited presence of interveners and regulatory bodies they actively contribute to spread misinformation about the virus*’ – KI 17, the Democratic Republic of the Congo

### Digital Adaptation

The survey data in this research found that many organisations faced challenges using digital solutions, encountering high levels of low digital literacy, difficulties keeping participants engaged, and lack of access to the internet or phones. The interview data shed more light on these issues and suggests significant differences in digitally adapting in the rural-urban divide. Some participants reported that digital approaches made it more challenging to engage with rural or more impoverished communities.

Local peace organisations have had to adapt projects to cope with the public health measures and respond to emerging needs. This research found that 40 percent of participants adapted projects using digital solutions, 22 percent radio, 21 percent face-to-face activities using personal protective equipment (PPE), 6 percent television, 3 percent interactive voice response (IVR) and 3 percent gaming for peace.

‘*While these tools are have evolved as part of our response to the current pandemic, we do believe that they would be useful in other situations that create limitations on face to face interaction. Adaptability is key to the success of any organisation. You have to mould your work according to the needs of the situation.’ –* KI 18, the Democratic Republic of the Congo

There was often a lack of digital infrastructure, technologies and skills in some communities where peacebuilding activities occurred. The financial cost of broadband and mobile services were also barriers to using the digital approach. Lack of infrastructures proved to be a significant issue in rural areas and countries experiencing more increased fragility. This existing divide explains why digital solutions were mainly concentrated in capital cities and remained marginal and non-relevant in some rural areas.

‘*Some areas don't have electricity connections, and most people would not have the Internet. So they don't have Internet, and some cannot even use it, and so it became difficult to use digital approaches. So, it became difficult to reach out to peopl*e’ – KI 13, CSO, Uganda

This was mainly expressed by participants from Sub-Saharan Africa who could not adopt digital solutions because of these connectivity issues.

‘*The internet-based tools are difficult to administer because of connectivity challenges, particularly in remote areas and also the challenge of smartphones and digital literacy. In urban settings, online tools may be used as network is much better.’* – KI 24, Uganda

To cope with the lack of access and disengagement from communities, some organisations implemented a hybrid approach.

‘*Community members were scared about the virus and as they are not familiar with digital solutions initially disengaged from the activities we proposed and started to engage again once we implemented a blended approach combining face to face interactions with other solutions.’* – KI 23, Nigeria

Where digital solutions were not an option because of the challenges in accessing them, organisations had to innovate other ways to continue their peace initiatives. In one instance, an organisation in Uganda hung loudspeakers in trees and used them to broadcast messages, enabling them to continue engaging with the communities.

‘*We never before used megaphones. We decided now to mount the megaphones in high trees and then would broadcast a message. There would be a song at such, and such a time, there will be episodes of a play about conflict and peacebuilding…there would be announcements.*’ – K 13, CSO, Uganda

Radio also often became a primary way organisations tried to continue peacebuilding operations. Especially in rural areas, community radios have a strong network and a high level of penetration. However, increasing competition over radio airtime led to increased costs and decreased access to space on the radio.

‘*Another constraint that emerged because of Covid was the use of digital approach is very expensive. Radio talk shows are quite popular here because I mean they can be listened to by anybody, even someone who has not gone to school. And then also eventually the prices were hiked but also in Uganda in particular, that was the time when we're holding up political campaigns for the general elections. However, during that time, radio stations became expensive. They were competing for the radio space to reach out to the public, and so, even if (redacted name of organisation) had money, probably there was no space on radio*.’ – KI 2, CSO, Uganda

The shift to the digital complicated these initiatives focusing on community relationships, dialogue and trust-building. Transforming relationships and building trust is essential in peacebuilding. Establishing relationships and mutual trust is nurtured by prolonged periods of dialogue, usually in person. COVID-19 public health restrictions, such as social distancing and travel restrictions, have meant peacebuilding efforts are being carried out using digital solutions or alternative formats (Alberti & Clark, 2020). The quantitative data showed that digital solutions helped build relationships and trust, with 22 per cent of participants strongly agreeing and 43 per cent agreeing that the adaptive tools they used helped promote trust-building. Meanwhile, 16 per cent disagreed, and 2 per cent strongly disagreed. However, when conducting interviews, respondents from the Great Lakes region, where the most affected communities live in rural areas, highlighted that these communities are essentially digitally excluded and further marginalised by the growing gap between under-connected and hyper-digitised nations.

Many participants reported difficulties in building relationships and fostering trust without direct interactions. Even in countries where face-to-face meetings were allowed, community members and mobilisers were reluctant to continue in-person activities. As a result, outreach reduced, and alternative solutions became a more dominant way to disseminate messages and engage with communities. Removing face-to-face interactions from peacebuilding processes often made it more challenging to build trust and relationships

‘*Face to face interactions with the communities we work with is key to building trust. Many are from the older generation and don't appreciate online interactions*.’ – KI20, Burundi

### Funding challenges

The pandemic is having significant effects on peacebuilding funding, with traditional donors directly affected. Donor-states are facing increasing internal budgetary and capacity constraints (Quaker United Nations Office, 2020, p. 3). Interactions between donors and peacebuilding organisations are raising concerns about post-pandemic funding. Many participants found donors inflexible in redirecting funding to implement projects that met the emerging needs of beneficiaries’ during the pandemic. A peacebuilding organisation in Lebanon reported having to use their organisation's reserve funds to pay for the new projects to meet the community's needs.

Many participants found donors inflexible in redirecting funding to implement projects that met the emerging needs of beneficiaries' during the pandemic.

‘*…it was not easy with the donor because we had to use our own funds to do this because the donor preferred to keep things extremely strict… So we decided to use our own reserves because the families were clearly expressing what they needed, and this was a priority for us*.’ – KI 6, CSO, Lebanon

The renewed ability of the organisation to meet the new needs of beneficiaries increased trust. Changing their priorities to providing food and sanitary products changed their relationship with the communities where they worked.

‘*But it changed our relationship with the families drastically because they saw that we could go beyond what's written in a project… And we really felt that the families were very appreciative of the fact that we had this flexibility*.’ – KI 6, CSO, Lebanon

While local actors understood that donors were also facing difficulties, they expressed frustration over donors reducing funding for non-COVID-19 related activities, including their support to cover their administrative work and having a limited budget resulted in financial challenges for some organisations.

‘*Also challenge was the donors’ side, and the donors themselves felt that you know we had some other donors who had scaled down—what, which again is understandable. But again, if the donor says we're reducing funding because of covid. Remember the other cost for (redactedorganisation name) has to incur the staff administration costs.*’ – KI 13, CSO, Uganda

A small number of participants reported that their donors were open to redirecting funds to more pertinent projects arising from COVID-19.

‘*In fact, they actually came to us saying if you need some flexibility with how you want to spend the money, please let us know, and we were able to reformulate.*’ – KI 7, CSO, Lebanon

However, even when donors were open to refocusing funds, some participants still faced challenges adjusting projects to meet their local organisations’ and donor requirements.

‘*So if you have money for ten public meetings and they tell you to adjust your programs and budgets, most likely from that money will not all be used because the numbers of people will substantially reduce. And then the donor wouldn't say have so many of those smaller engagements, but also because of the time, because you cannot have so many meetings of fewer people, and the donors were not willing to let you, you know, spend all that money, or maybe try to adjust so they had some limits*.’ – KI 13, CSO, Uganda

Additionally, while some were open to reorienting funding, they often did not consider extra costs such as masks and hand sanitisers, considering those as part of the regular cost of projects, leaving less money for peacebuilding activities.

‘*Donors requested us to conduct meetings with a reduced number of people, but no additional budget for face masks and installation of handwashing points was provided. This was a challenge, especially at the beginning when it was difficult to find these items on the market.*’ – KI 17, the Democratic Republic of the Congo

### Building peace in the new normal

The survey and interview data indicate organisations are facing challenges during the pandemic. At the same time, it has been a learning opportunity for peacebuilding actors. Sixty-eight percent of respondents said they would continue incorporating alternative solutions into peacebuilding interventions after the pandemic, and 24 percent said they would not.

‘*Working during the COVID-19 pandemic has been a learning experience. As we had limited access to the field, we were forced to use the community radio more. While at the beginning we were worried we would not have reached as many people as we usually did, we realised that sharing short messages and jingles on peacebuilding related issues had a bigger outreach, and we would like to continue this type of activities when we will be able to work again face to face.'- KI 19, Chad*

‘*In peacebuilding, it is hard to use digitalised materials, especially when doing mediation and dialogue. I prefer using the old methods of face to face dialogue or mediation.’* – KI 25, Rwanda

While the respondents expressed a desire and need to go back to face-to-face interactions and highlighted that remote solutions are limiting the impact and capacity to operate in rural areas, they acknowledged that some adaptive solutions improved the quality of interventions. Some improvements include increased engagement of national actors linking micro and macro levels of actors.

‘*As part of our adapted interventions, we set up a series of online dialogue sessions and round tables on different peacebuilding related topics. While we realised that we were missing out on rural communities, we registered an increased engagement of national elites in our activities. While before we were engaging mainly with local actors, now we have a space to discuss even with national-level actors that in many cases are the ‘mind’ behind local conflicts in our country.’* – KI 23, Nigeria

Incorporating digital solutions during COVID-19 allows organisations to use a hybrid model after the pandemic, which many participants said they would do.

‘*We now include people of a wider area than we could before covid, so to keep those people engaged, we plan to use hybrid meeting methods that include both in-person and video participation.’* – KI 17, DRC

### Lessons learned and the way forward

The COVID-19 pandemic has forced peacebuilders to adapt. This shift raises questions about what is needed to carry out peacebuilding efforts after the pandemic. Three primary considerations arise from the research findings.

First, the results suggest that the pandemic created more support for localisation. Over the past 20 years, localisation has been at the centre of debate but was never implemented globally. Travel restrictions force larger organisations to rely on smaller ones and local CSOs with a strong presence and acceptance from the communities. CSOs had better access to communities during the pandemic and became the primary actors carrying out peacebuilding interventions. With external actors mainly excluded from frontline processes and local actors carrying out the projects, localisation should be emphasised beyond the current practice of contracting local organisations as implementing partners. This shift may enable the implementation of adaptive peacebuilding interventions where interveners support communities and local actors to build their capacity and resilience to self-organise and cope with future crises (de Coning, 2018, p. 307).

Based on the emerging needs from this renewed push for localisation, this research highlights structural issues. Existing funding mechanisms are locked into pre-established results and have minimal space for programmatic and operational adjustments. The survey and interview data shows a need for donors and funders to be more flexible, especially in times of crisis. This flexibility would significantly help in sustaining financial support for peacebuilding organisations. It is essential to shift funding mechanisms based on accountability that strictly evaluates peace interventions based on pre-determined results toward a system focusing on the outcome rather than on the outputs of the interventions. For this to happen, donors need to rethink their funding strategy to create adaptive oriented funding schemes.

Moreover, donors need to look into the work done by local organisations and consider rethinking how their funding is allocated. Currently, local organisations primarily receive funding from more prominent NGOs, INGOs or International Organizations with a limited presence on the ground and subcontract them to carry out the activities in the field. Investing in increasing the capacity of local organisations and channelling funding directly to local entities would reduce the overhead costs of interventions and provide more resources for the work on the ground.

A third consideration arising from this research is how peacebuilding activities will function when normal activities return. During the pandemic, many organisations adopted blended solutions, incorporating face-to-face and remote approaches. However, digital and remote solutions are not suitable for hard-to-reach areas, resulting in increasing inequalities. If these communities were to become digitally connected, there would need to be significant investment in digital technologies, digital infrastructure, and digital skills and building capacity and community acceptance. As a result, using these solutions raises several questions about the inclusivity and cultural appropriateness of the interventions and might be posing challenges in building trust with communities. On the other hand, the alternative solutions improved the quality and outreach of interventions and should not be discontinued once organisations return to the ‘*old normal’*. As shown in this research, these alternative solutions digital solutions allow for new relationships and connections between micro and macro-level actors. The use of remote and digital solutions provided a new way to connect local peacebuilding initiatives to the national level, creating a space for national elites to hear local actors.

Digital and remote solutions are not suitable for hard-to-reach areas, resulting in increasing inequalities.

To allow peacebuilding actors to continue implementing these adaptive solutions and continue sustaining the localisation process, donors and international organisations must reconsider how funding is allocated and administered. Without a change in policies regulating how organisations can manage received funding and how funding reaches local organisations, there may be issues at different levels that will not be able to capitalise on the lessons learned through this experience.

This study is the first step in understanding how COVID-19 impacts peacebuilding in the present and how it will change peacebuilding operations in the future. The challenges and opportunities for peacebuilding arising during the pandemic will inform how peacebuilding will function in the future. Further investigations are needed after the pandemic to fully understand the story to see if and how peacebuilding changes in the medium to long term.
